# Coaching junior faculty for the uncertainties of academic professional practice

**DOI:** 10.5116/ijme.613c.6f82

**Published:** 2021-09-30

**Authors:** Richard McKnight, Dimitrios Papanagnou

**Affiliations:** 1 Department of Emergency Medicine, Sidney Kimmel Medical College at Thomas Jefferson University, Philadelphia, PA, USA

**Keywords:** Coaching, junior faculty, uncertainties, academic professional practice

## To the Editor

Physicians beginning a career in academic medicine typically face a host of challenges, both professional and personal. The transition to independent clinical practice from graduate training (i.e., residency) requires learning rapidly in order to flourish in an organizational setting. This is also when graduates must design a career path that plays to their strengths and interests and build a supportive, collegial network. Layered on top of all this are the demands of adult development, which require forging a personal identity (e.g., becoming a spouse or partner, having children, buying a home). We posit that coaching, which has been traditionally reserved for middle- and senior-level personnel, as well as leadership training, has the potential to equip junior faculty with skills needed to navigate the uncertainty associated with the transition into academic, professional practice.

Junior faculty experience high stress during their transition to independent professional practice, particularly as they navigate areas of uncertainty following heavily scaffolded training. Most academic faculty perform a variety of roles, including clinician, educator, researcher, and/or administrator. Recent data from the Association of American Medical College (AAMC) StandPoint Faculty Engagement Survey highlighted that close to 30% of faculty respondents from US medical schools experience one or more symptoms of burnout.^1^ There is a need to improve faculty wellness, particularly for junior faculty. In particular, burnout is increasingly prevalent in emergency medicine (EM) providers.^2^ Therefore, it is essential to equip faculty, specifically junior faculty, with resilience skills that would contribute to living happier lives and continuing in their specialty longer.^2^

Viggiano and Strobel have articulated a faculty life cycle model that addresses the challenges of transitioning to the junior faculty role.^3^ In the first two phases of their model, i.e., Recruitment and Orientation, they define the need for academic departments and new faculty members to align values, needs, goals. When this does not occur, retention and engagement both become problems. Several innovations have been cited that address the needs of junior faculty, but most focus on hiring processes. They do not take a holistic approach that addresses role integration and adult development.^4^   We posit that an intensive coaching program for junior faculty, which provides them with a coach to work on role performance and adult development, has the potential to address these issues. Coaching in academic medicine has the potential to improve resilience to stress, hone non-technical—typically interpersonal—skills, and enhance technical and procedural skills.^5^ We wanted to determine if a faculty coaching program for junior faculty members in our Department of EM was feasible (i.e., would faculty make the commitment and meaningfully engage in a coaching program?), and identify areas junior faculty struggle with early in their careers and the adjustments they have to make as faculty.

To examine the feasibility, we recruited fourteen volunteer coaches (seven men, seven women) with extensive training and experience with coaching. None were formally affiliated with or employed by our institution, and all were professional colleagues of the authors. The coaches were motivated to volunteer because of the novel experience it presented. Eleven coaches were experienced business coaches; two were psychologists, and one was a recently retired pediatrician. We defined junior faculty as those who were within five years of graduating from residency training. Seven male and seven female junior faculty were enrolled in our program. Each faculty member completed a pre-coaching questionnaire inquiring about their personal goals. Additionally, faculty were asked to complete a 30-45-minute telephone interview with the coaching director to assure fit with the program and matching with a coach.

We conducted a 2-hour in-person meeting for the coaches in November 2019, which was then followed by an opportunity for them to introduce themselves to the junior faculty. The first 90-minute coaching session was conducted during this meeting. The program required at least one monthly face-to-face or telephone meeting of 60-90 minutes. In most cases, particularly after the onset of the COVID-19 pandemic, meetings were conducted virtually, either through Zoom or FaceTime. On average, coaches and clients met 1.5 times per month.

During the early stages of the program, we emphasized resilience skills as the primary outcome; but during the pre-program interviews with the faculty, we discovered a need for outcomes associated with coaching in most workplaces (i.e., navigating organizational dynamics, making career decisions, enhancing interpersonal skills). Several faculties requested help with stress and burnout. Specifically, pre-program interviews with faculty revealed several workplace concerns, including decision fatigue; being perceived as needy or vulnerable; the ability to get promoted; the need to speak with someone regularly to process on-the-job stressors; and reconciling ways to advocate for themselves without being perceived as negative or disgruntled. We also discovered goals that are germane to early adulthood, such as balancing dedication to work with beginning a marriage, purchasing a home, or becoming a new parent. One faculty member shared,

"I'm a people pleaser, sometimes to the extent of not reaching a goal. I need to be more assertive. I'm limited in my ability to say no. coaching could help me."

These observations were helpful in preparing coaches to deal with both on- and off-the-job issues. For purposes of reflecting on interpersonal skills, we employed two psychological instruments. Coaches spent at least one session interpreting the results of each. The Dominance, Influence, Steadiness and Conscientiousness (DiSC) tool was used for the reflection of faculty members' interpersonal communication styles, strengths, and limitations.^6^ We also administered the Emotional Quotient Inventory (EQ-I 2.0), an instrument to guide conversations surrounding emotional intelligence.^7^ During the first six months, coaches worked with faculty to set goals for the duration of coaching. The remaining six coaching sessions were focused on coaching towards these goals.

The COVID-19 pandemic began three months into our program. We were unsure if we would continue the program due to distractions and scheduling, but a survey of both coaches and clients showed a high interest in continuing the program. In some cases, during March and April 2020, the frequency of in-person meetings slowed, and all contact was via Zoom or FaceTime, but the process continued, and faculty appreciated the support coaches provided in handling the added stress of the pandemic. Faculty enrolled in the program commented on improved job satisfaction. Faculty turnover in this cohort was minimal. Faculty credited their coach for helping them work through organizational problems inhibiting job satisfaction. One faculty member shared, "Were it not for this program, and I may have considered leaving. My coach helped me find ways to make the environment work for me." Other benefits reported included gaining self-confidence, managing stress incurred by the pandemic; learning how to advocate for oneself; reflecting on interpersonal interactions with work colleagues, and understanding the complex culture of the clinical workplace.

We were fortunate to have volunteer coaches during the program's first iteration, but the program will not be sustainable with volunteer coaches. The authors are currently seeking support to continue the program. They are considering a program variation in which late-career physicians (perhaps alumni of the medical school or the residency program) are trained to serve as coaches. We have learned that one-on-one coaching is feasible in junior faculty and may be a considered intervention to support them further as they transition into professional practice.

### Conflicts of Interest

The authors declare that they have no conflict of interest.
